# The Effect of Host Immunity on Predicting the Mortality of Carbapenem-Resistant Organism Infection

**DOI:** 10.3389/fcimb.2020.00480

**Published:** 2020-09-10

**Authors:** Qun Lin, Yue Wang, Ying Luo, Guoxing Tang, Shusheng Li, Yicheng Zhang, Liyan Mao, Weiyong Liu, Feng Wang, Ziyong Sun

**Affiliations:** ^1^Department of Laboratory Medicine, Tongji Medical College, Tongji Hospital, Huazhong University of Science and Technology, Wuhan, China; ^2^Department of Emergency Medicine, Tongji Medical College, Tongji Hospital, Huazhong University of Science and Technology, Wuhan, China; ^3^Department of Haematology, Tongji Medical College, Tongji Hospital, Huazhong University of Science and Technology, Wuhan, China

**Keywords:** carbapenem-resistant organisms (CROs), IFN-γ^+^CD4^+^ T cell number, predictive model, lymphocyte number, lymphocyte function

## Abstract

Carbapenem-resistant organisms (CROs) are associated with considerable mortality clinically. There is a lack of effective tool to predict individual prognosis. We aim to determine if host immunity can be utilized to predict the prognosis of patients infected with CRO. From December 2018 to August 2019, we recruited CRO-infected patients to evaluate risk factors for 30-day mortality. Clinical, routine laboratory, immune and microbiological features were investigated and subjected to univariate and multivariate analyses. The final predictive models were established based on the regression coefficients of multivariate logistic regression. A total of 127 CRO-infected patients were enrolled in our study, including 85 survivors and 42 non-survivors. The number and IFN-γ producing ability of lymphocytes were remarkably decreased in non-survivors. The number of IFN-γ^+^CD4^+^ T cells could effectively predict 30-day mortality of CRO infection. Its area under the receiver operating characteristic (ROC) curve, sensitivity, specificity and accuracy, were 0.889 (95% confidence interval [CI], 0.834–0.945), 81.0, 80.0, and 80.3%, respectively. In multivariate analysis of laboratory parameters, IFN-γ^+^CD4^+^ T cell number and creatinine concentration were selected for the 2-marker model to predict prognosis fleetly. Its area under the ROC curve, sensitivity, specificity and accuracy were 0.894 (95% CI, 0.841–0.947), 83.3, 82.4, and 82.7%, respectively. Impaired lymphocyte function was an important factor to affect the outcome of CRO-infected patients. A 2-marker model based on the combination of IFN-γ^+^CD4^+^ T cell number and creatinine showed good performance in predicting the prognosis of CRO infection.

## Introduction

Over the last decade, carbapenem-resistant organisms (CROs), mainly including carbapenem-resistant *Enterobacteriaceae* (CRE), carbapenem-resistant *Acinetobacter baumannii* (CRAB) and carbapenem-resistant *Pseudomonas aeruginosa* (CRPsA), have caused serious healthcare-associated infections and disseminated globally (Kizny Gordon et al., 2017; Tomczyk et al., 2019). CROs have been highlighted as critical pathogens in the World Health Organization (WHO) prioritization of pathogens (World Health Organization, 2017). Due to multiple and high levels of antimicrobial resistance, these bacteria have limited therapeutic options in clinical and resulted in an increased risk of mortality in patients (Falagas et al., 2014; Lemos et al., 2014; Zhang et al., 2016; Agyeman et al., 2020). To identify those at high risk of poor prognosis, it is important to develop effective predictive tools, which may promote a shift from empirical treatment to precision-guided therapy tailored to each patient with expected improved patient outcomes.

Patients with immunosuppression are more likely to suffer opportunistic infection and poor prognosis (Zhu et al., 2018; Luo et al., 2019). Several reports have indicated that impaired host immunity is an independent risk factor for CRO infection and mortality (Huang et al., 2012; Miller and Johnson, 2016; Wang et al., 2016; Zhu et al., 2018). Currently, clinicians usually assess patients' immune function based on whether patients have underlying diseases, and this has lots of inaccuracy. Lymphocyte function is one of the most important characteristics to reflect host immunity; however, lymphocyte function assessment has not been widely used in clinical practice (Drabe et al., 2019; Luo et al., 2019).

Better understanding of variables that influence mortality is essential to improving outcomes in patients with CRO infection. There also have been several reports regarding the risk factors and their effects on CRO infection and mortality, such as, appropriate combination therapy, carbapenem MICs and carbapenemase (Patel and Nagel, 2015; Gutiérrez-Gutiérrez et al., 2017; Tamma et al., 2017; Wang et al., 2018; Lin et al., 2019). In this study, we obtained peripheral blood of CRO-infected patients and assessed host immunity based on assessment of lymphocyte number and function simultaneously. We also collected patients' clinical data, including demographics, vital signs, hematology and biochemistry, microbiology, diagnoses, hospital contacts, surgical procedures, antibiotic therapy and 30-day outcome. Based on these data, we developed mathematical models to predict inpatient mortality by multivariate logistic regression. To our knowledge, this is the first study combining host immunity and clinical data to establish predictive model to identify individuals at high risk of mortality in the early stage of CRO infection. This study not only provides an innovative insight into the characteristics of host immunity in CRO infection, but also offers tools to predict CRO-infected individual outcomes.

## Materials and Methods

### Participants

Between December 2018 and August 2019, based on microbiological results and clinical symptoms, suspected CRO-infected patients were consecutively recruited from Tongji Hospital, Tongji Medical College, Huazhong University of Science and Technology, which is the largest university-affiliated tertiary medical center in central China. All inpatients with bloodstream infection or respiratory tract infection triggered by carbapenem-resistant *Klebsiella pneumoniae* (CRKP) or CRAB were included. Carbapenem resistance was defined as resistance to either imipenem or meropenem. Patients with a blood culture positive were defined to bloodstream infection. Patients with positive cultures from respiratory were defined to respiratory tract infection according to the Center for Disease Control and Prevention (CDC) and National Healthcare Safety Network (NHSN) criteria (CDC/NHSN, n.d.).

Patients with CRO pneumonia was diagnosed by using a combination of imaging, clinical and laboratory criteria, as follows: (1) Imaging results, the patients met one of the following criteria: new, persistent, and progressive imaging abnormalities (infiltrate, consolidation or cavitation). In patients without underlying pulmonary or cardiac disease, one imaging test result is acceptable. In patients with underlying pulmonary or cardiac disease, two or more serial chest imaging test results are needed. (2) Signs/symptoms, the patients met one of the following criteria: (a) fever (>38.0°C), (b) leukopenia (≤ 4,000 WBC/mm^3^) or leukocytosis (≥12,000 WBC/mm^3^), and (c) altered mental status with no other recognized cause in adults ≥70 years old. And, the patients met one of the following criteria: (a) new onset of purulent sputum or change in character of sputum, or increased respiratory secretions, or increased suctioning requirements; (b) new onset or worsening cough, or dyspnea, or tachypnea; (c) rales or bronchial breath sounds; and (d) worsening gas exchange. (3) Laboratory, CRO was isolated and identified from blood, sputum or bronchoalveolar lavage. The exclusion criteria of the patients were as follows: (1) having no typical imaging abnormalities; (2) having no typical clinical signs and symptoms of pneumonia; (3) missing the results of the number and function of lymphocytes; (4) loss to follow-up.

This study was approved by the ethical committee of Tongji Hospital, Tongji Medical College, Huazhong University of Science and Technology. All participants gave written consent to the study.

### Data Collection and Definition

At the time of notification of a positive culture, all suspected patients' peripheral blood samples were collected to perform lymphocyte function assay as described as the following methods. Relevant laboratory results and clinical features of the day of positive culture collection were documented. The worst value within 24 h of the positive culture collection was selected as the Acute Physiology and Chronic Health Evaluation II (APACHE II) score. Moreover, other information and data were collected, including demographic, history of transferring from other institutions or wards prior to a positive culture (≤ 30 days), CRE colonization (within 30 days), underlying diseases, and subsequent medication or intervention therapy after the positive culture. The clinical outcome in this study was 30-day mortality measured from the day of first positive culture collection.

Isolates from cultures that had been collected more than 48 h after admission to hospital were defined as “hospital-acquired infection.” Mixed infections were defined as more than one pathogen isolated from the same infection site within 3 days (Wang et al., 2016; Liang et al., 2018). Antibiotics to which the isolate was susceptible based on antibiotic susceptibility testing was defined as active drugs. Appropriate therapy was defined as treatment provided with at least one active drug within 5 days of the time the first positive culture was obtained and maintained for at least 48 h (Gonzalez-Padilla et al., 2015; Gutiérrez-Gutiérrez et al., 2017). If the active drug was started in 2 days or sooner, we considered it early appropriate therapy. If the regimen was changed during the course, we considered the antibiotic regimen as the one started in the 5 days or sooner period after infection and administered for at least half of the duration of therapy. Appropriate combination therapy was defined as 2 or more appropriate antibiotics.

### Microbiologic Methods

Bacterial species were identified using matrix-assisted laser-desorption ionization time-of-flight mass spectrometry (Bruker Daltonics Inc., Billerica, Massachusetts). Antibiotic susceptibility testing was performed *in vitro* by using the Kirby-Bauer disk diffusion method according to the standards set by the Clinical Laboratory Standards Institutes. Carbapenem (meropenem and/or imipenem) resistance was confirmed by the E-test method (bioMérieux, Durham, North Carolina), according to the manufacturer's instructions (AB Biodisk, Solna, Sweden). CRE isolates were tested for carbapenemase genes (*bla*_KPC_, *bla*_NDM_, *bla*_IMP_, *bla*_VIM_ and *bla*_OXA−48_) by polymerase chain reaction (PCR).

### Assessment of the Number and Function of Lymphocytes

We performed PMA/ionomycin-stimulated lymphocyte function assay as described previously (Hou et al., 2018; Luo et al., 2019). In brief, the procedures are shown as following: (1)100 μl of whole blood was diluted with 400 μl of IMDM medium in polystyrene round-bottom tubes with caps (Falcon 352054, Becton Dickinson); (2) the diluted whole blood was incubated in the presence of Leukocyte Activation Cocktail (Becton Dickinson GolgiPlug™) for 4 h at 37°C; (3) 300 μl of supernatant was aspirated, then cell suspensions were labeled with five kinds of antibodies (anti-CD45-PerCp, anti-CD3-FITC, anti-CD4-APC/Cy7, anti-CD56-PE/Cy7, and anti-CD8-PE) (Becton Dickinson) and incubated for 15 min at room temperature; (4) the cells were fixed and permeabilized subsequently; (5) the cells were stained with intracellular anti-IFN-γ-APC antibody; and (6) the cells were analyzed with FACSCanto flow cytometer (Becton Dickinson). The percentages of IFN-γ^+^ cells in different cell subsets were defined as the function of them, and the flow analysis template is shown in Supplementary Figure 1.

The total number of lymphocytes in peripheral blood was counted by hemocytometer. The absolute numbers of different lymphocyte subsets were calculated by multiplying the percentages with total lymphocyte count as follows: CD4^+^ T cell count = total lymphocyte count × CD3^+^CD4^+^CD8^−^%; CD8^+^ T cell count = total lymphocyte count × CD3^+^CD4^−^CD8^+^%; NK cell count = total lymphocyte count × CD3^−^CD56^+^%. The percentages of CD3^+^CD4^+^CD8^−^, CD3^+^CD4^−^CD8^+^, and CD3^−^CD56^+^ cells in total lymphocytes were obtained as above lymphocyte function assay. The absolute numbers of different functional lymphocyte subsets were calculated as follows: IFN-γ^+^CD4^+^ T cell count = CD4^+^ T cell count × IFN-γ^+^CD4^+^ T cells %; IFN-γ^+^CD8^+^ T cell count = CD8^+^ T cell count × IFN-γ^+^CD8^+^ T cells %; IFN-γ^+^ NK cell count = NK cell count × IFN-γ^+^ NK cells %.

### Statistical Analysis

Continuous variables were compared with the Student's *t*-test (for normally distributed variables) or the Mann–Whitney U test (for non-normally distributed variables) and presented as the mean ± standard deviation (SD) or median. Categorical variables were assessed using the χ^2^ test or Fisher exact test and were presented as percentages. Combined with professional knowledge, variables with a *P* < 0.05 in univariable analyses were included in the further multivariable logistic regression analyses to determine the independent variables that were associated with mortality. Then the regression equation (predictive model) was obtained and a score for each patient was calculated. Receiver operating characteristic (ROC) curve analysis was performed on these scores and factors to assess the ability and the cut-off values. Area under the curve (AUC), optimal combination of sensitivity and specificity, positive predictive value (PPV), negative predictive value (NPV) and accuracy were reported, as well as the 95% confidence intervals (CI) of the AUC. Kaplan-Meier curves were compared by using log-rank tests. The independent impact of host immunity on survival of CRO infection was evaluated by a propensity score weighting method.

Graphpad Prism 5.01 (GraphPad Software, CA, USA) was used for plotting the data. SPSS software (version 22.0) and R4.0.2 were used in statistical analysis. Statistical significance was determined as *P* < 0.05.

## Results

### Study Population

**Figure 1:**
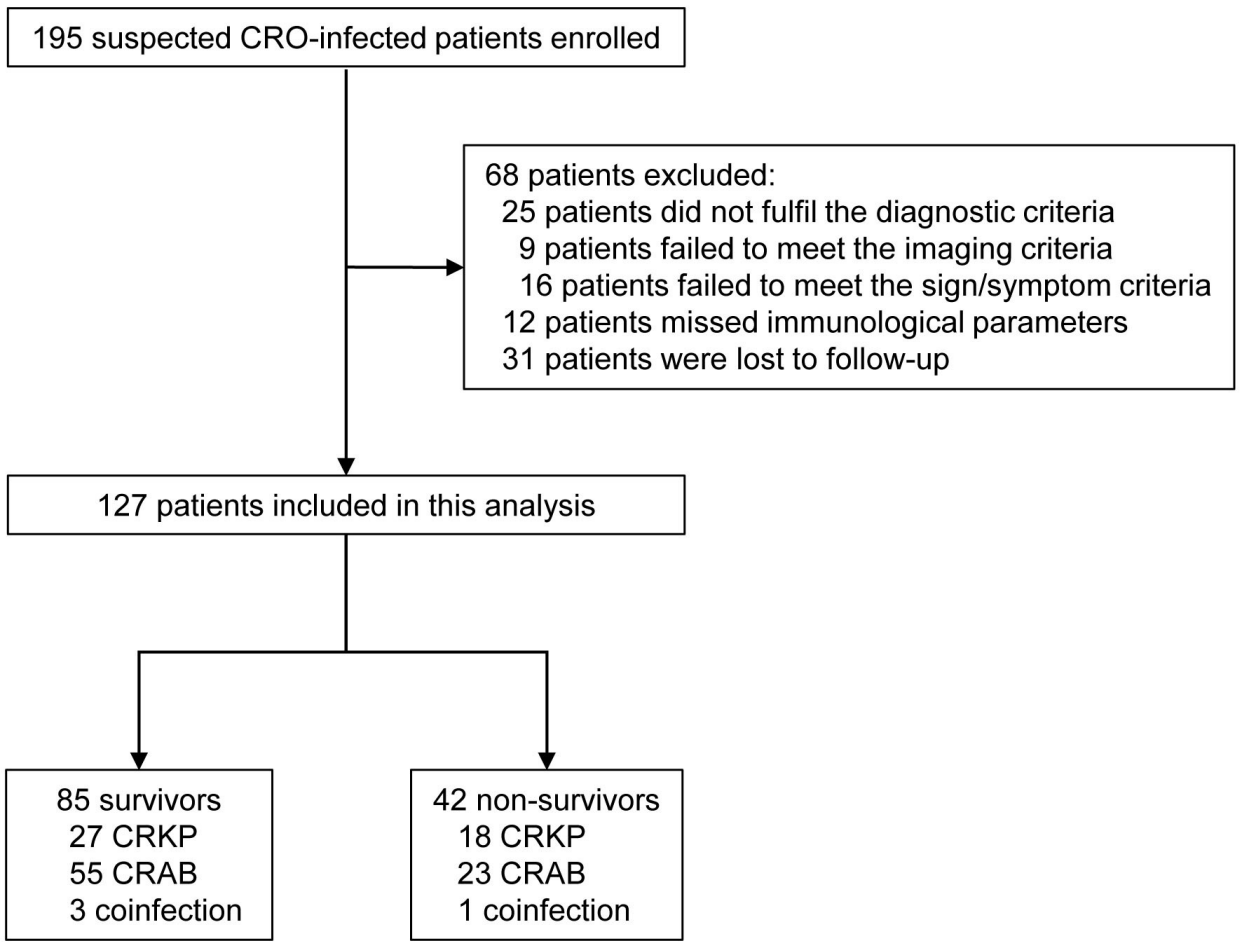
Flow chart of included patients with CRO infection. CROs, carbapenem-resistant organisms; CRKP, Carbapenem-resistant *Klebsiella pneumonia;* CRAB, Carbapenem-resistant *Acinetobacter baumannii*.

A total of 195 suspected cases were enrolled in this study. There were 68 patients excluded from the study: 25 patients did not fulfill the diagnostic criteria (9 patients failed to meet the imaging criteria and 16 patients failed to meet the sign/symptom criteria); 12 patients missed immunological parameters because of no enough lymphocytes collected for performing lymphocyte function assay; 31 patients were lost to follow-up (discharging from our hospital within 30 days after diagnosis of CRO infection and failing to follow-up because of non-compliance by telephone). Ultimately, data for 127 CRO-infected patients were included in the analysis after applying inclusion and exclusion criteria (Figure 1). The ages of patients ranged from 19 to 94 years, with a median age of 54 years, and 99 patients (77.95%) were male. The 30-day mortality rate was 33.07% and the overall in-hospital mortality rate was 14.96%. After exclusion of 68 patients, 127 patients with definite outcome were finally enrolled, including 85 survivors (66.93%) and 42 non-survivors (33.07%). The risk factors for 30-day mortality among patients with CRO infection were listed in Table 1.

**Table 1:** Baseline characteristics of CRO-infected patients stratified by 30-day mortality.

**Characteristic**	**Survivors (*n* = 85)**	**Non-survivors (*n* = 42)**	***P*-Value**
Age (years)	52.13 ± 14.40	55.26 ± 14.20	0.249
Male sex	62 (72.9)	37 (88.1)	0.053
Hospital-acquired infection	75 (88.2)	38 (90.5)	0.938
APACHE II score	15.34 ± 5.64	16.21 ± 6.90	0.448
Transferring wards during hospitalization^a^	45 (52.9)	24 (57.1)	0.655
History of prior hospitalization^a^	65 (76.5)	29 (69.0)	0.369
ICU admission^a^	33 (38.8)	21 (50.0)	0.231
Surgery^a^	56 (65.9)	25 (59.5)	0.483
CRE colonization^a^	7/35 (20.0)	6/19 (31.6)	0.342
Immunosuppressive agents^a^	6 (7.1)	5 (11.9)	0.361
**Location at time of culture**			
Intensive care unit	27 (31.8)	13 (31.0)	0.074
Medical ward	33 (38.8)	9 (21.4)	
Surgical ward	25 (29.4)	20 (47.6)	
**Culture sources**			
Blood	13 (15.3)	11 (26.2)	0.313
Sputum	56 (65.9)	23 (54.8)	
Broncho-alveolar lavage	16 (18.8)	8 (19.0)	
**Microbiology**			
*Klebsiella pneumoniae*	27 (31.8)	18 (42.9)	0.465
*Acinetobacter baumannii*	55 (64.7)	23 (54.8)	
Polymicrobial^b^	3 (3.5)	1 (2.4)	
Concomitant infection by other sources	7(8.2)	1 (2.4)	0.374
**Concomitant infection by other pathogens**^**c**^	34 (40.0)	23 (54.8)	0.116
*Staphylococcus aureus*	3	1	
*Pseudomonas aeruginosa*	7	4	
*Stenotrophomonas maltophilia*	2	4	
Candida species	13	12	
Others	11	3	
**Carbapenemase genes**			
*bla*_KPC_	30 (35.3)	19 (45.2)	0.279
*bla*_OXA−48_	58 (68.2)	24 (57.1)	0.219
**Comorbidities**			
Pulmonary disease	48 (56.5)	26 (61.9)	0.559
Cardiovascular disease^d^	49 (57.6)	28 (66.7)	0.328
Neurologic disease	59 (69.4)	15 (35.7)	<0.001
Gastrointestinal disease	24 (28.2)	10 (23.8)	0.596
Hepatobiliary disease	23 (27.1)	18 (42.9)	0.073
Renal disease	15 (17.6)	16 (38.1)	0.012
Diabetes mellitus	15 (17.6)	5 (11.9)	0.403
Malignancy	8 (9.4)	5 (11.9)	0.663
Immune rheumatic disease	4 (4.7)	1 (2.4)	1.000
Multiple trauma	19 (22.4)	5 (11.9)	0.157
**Invasive procedures or treatments**^**a**^			
Peripherally Inserted Central Catheter	71 (83.5)	36 (85.7)	0.750
Tracheotomy	67 (78.8)	37 (88.1)	0.202
Bronchofiberscope	73 (85.9)	39 (92.9)	0.393
Urinary catheter	71 (83.5)	29 (69.0)	0.061
Nasogastric tube	56 (65.9)	22 (52.4)	0.141
Puncture or biopsy	12 (14.1)	7 (16.7)	0.705
Surgical drainage	69 (81.2)	33 (78.6)	0.728
Renal replacement therapy	23 (27.1)	27 (64.3)	<0.001
Multiple organ failure	27 (31.8)	29 (69.0)	<0.001
**Antibiotic therapy**			
Appropriate therapy	59 (69.4)	35 (83.3)	0.092
Early appropriate therapy	39 (45.9)	25 (59.5)	0.148
Appropriate monotherapy	42 (49.4)	23 (54.8)	0.570
Appropriate combination therapy	17 (20.0)	12 (28.6)	0.279
**Biological parameters**			
WBC counts (× 10^9^/L)	10.36 (7.36–14.23)	13.79 (10.18–17.28)	0.002
Neutrophils proportion (%)	81.60 (74.80–88.75)	90.70 (85.55–92.93)	<0.001
Lymphocyte counts (× 10^9^/L)	1.08 (0.77–1.79)	0.57 (0.34–0.79)	<0.001
Hemoglobin (g/L)	95.00 (81.00–110.50)	93.00 (78.75–107.00)	0.289
Platelet counts (× 10^9^/L)	189.00 (124.50–283.00)	94.00 (46.00–195.50)	<0.001
ALT (U/L)	22.00 (14.00–44.50)	27.50 (15.00–87.00)	0.181
AST (U/L)	28.00 (22.00–43.00)	37.00 (24.00–89.75)	0.030
Albumin (g/L)	31.60 (28.65–36.70)	32.80 (27.13–36.40)	0.762
Creatinine (μmol/L)	56.00 (40.50–84.50)	84.00 (48.75–114.00)	0.010
**Immunology parameters**			
CD4^+^ T cells (/μl)	412.00 (251.50–660.50)	154.50 (72.25–277.50)	<0.001
CD8^+^ T cells (/μl)	236.00 (149.50–416.50)	97.00 (56.75–162.50)	<0.001
NK cells (/μl)	78.00 (39.00–162.00)	26.00 (14.75–58.25)	<0.001
IFN-γ^+^ CD4^+^ T cells (%)	21.25 (14.60–28.29)	12.79 (7.49–20.55)	<0.001
IFN-γ^+^ CD8^+^ T cells (%)	55.17 (38.20–67.83)	42.22 (20.53–63.97)	0.023
IFN-γ^+^ NK cells (%)	55.70 (33.77–81.43)	45.11 (22.89–68.60)	0.028
IFN-γ^+^ CD4^+^ T cells (/μl)	75.00 (45.00–141.00)	19.50 (7.50–36.25)	<0.001
IFN-γ^+^ CD8^+^ T cells (/μl)	107.00 (61.50–191.50)	35.00 (15.75–70.75)	<0.001
IFN-γ^+^ NK cells (/μl)	39.00 (16.00–72.00)	11.50 (5.00–19.25)	<0.001

*Data are presented as means ± SD, medians (25th-75th centiles) or number (percentage)*.

*APACHE II, Acute Physiology and Chronic Health Evaluation II; ICU, intensive care unit; CRE, carbapenem-resistant Enterobacteriaceae; KPC, K pneumoniae carbapenemase; OXA, oxacillinase; WBC, white blood cells; ALT, alanine aminotransferase; AST, aspartate aminotransferase; IFN-γ, interferon-γ; NK, Natural killer cell*.

a *within 30 days of collection of the first positive culture*.

b *patients coinfected with A. baumannii and K. pneumoniae*.

c *including patients coinfected with A. baumannii and K. pneumoniae*.

d *including hypertension*.

### Univariate Analysis of Risk Factors for 30-Day Mortality

The most frequent cases were pneumonia (81.10%, 103/127) and bacteremia (18.90%, 24/127). CRAB accounted for the predominant infections (61.42%, 78/127) and CRKP accounted for 35.43% (45/127). 57 cases were concomitant infection, thereinto, 4 patients coinfected with CRAB and CRKP. All isolates were carbapenemase-producing strains. No significant differences were observed between the 2 groups in infection sources, causative bacteria and carbapenemase-producing status.

Age, gender, infection type, APACHE II score, transferring wards during hospitalization, history of prior hospitalization, ICU admission, surgery, CRE colonization and receipt of immunosuppressive agents in the prior 30 days were not associated with 30-day survival in univariate analysis. Neurologic disease, as a complication, was much more frequent among survivors (*P* < 0.001), along with renal disease in contrary (*P* = 0.012). No significant differences were noted in the remaining comorbidities and invasive procedures. Multiple organ failure (MOF) and dialysis were more common in non-survivors compared with survivors (*P* < 0.001). A total of 94 patients (74.02%) received appropriate therapy in our study, including 65 patients who received monotherapy (87.84%, 65/74) and 29 who received combination therapy (30.85%, 29/94). In addition, 64 patients (50.39%) received early appropriate therapy. There was no significant difference in antibiotic therapy (Table 1).

**Figure 2:**
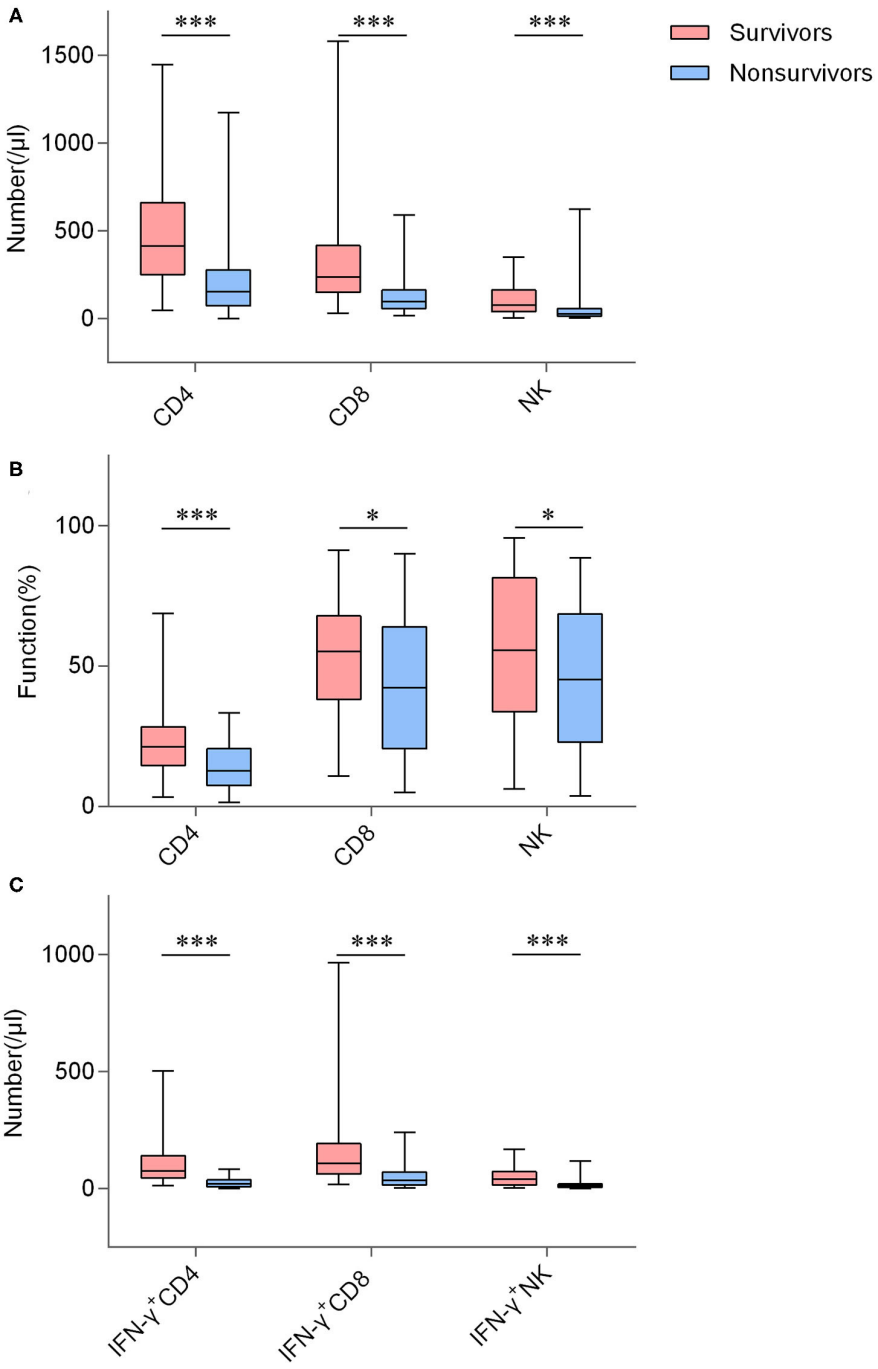
The number and function of lymphocyte in CRO-infected patients with different outcomes. **(A)** Boxplots showing the number of CD4^+^ T cells, CD8^+^ T cells, and NK cells; **(B)** Boxplots showing the function of CD4^+^ T cells, CD8^+^ T cells, and NK cells; **(C)** Boxplots showing the number of IFN-γ^+^CD4^+^ T cells, IFN-γ^+^CD8^+^ T cells, and IFN-γ^+^ NK cells. *P < 0.05, ***P < 0.001 (Mann–Whitney *U*-test). The boxplots depict the median, and 25th to 75th percentiles, and the whisker indicates the maximum and minimum values.

There were statistical differences between the two groups in terms of white blood cell (WBC) counts (*P* = 0.002), neutrophils proportion (*P* < 0.001), lymphocyte counts (*P* < 0.001), platelet counts (*P* < 0.001), aspartate aminotransferase (AST) concentration (*P* = 0.030), and creatinine concentration (*P* = 0.010). Whereas, hemoglobin, albumin and alanine aminotransferase were not different in concentration. Figure 2 shows detailed lymphocyte characterizations of CRO-infected patients with different outcomes. Number and function (IFN-γ producing ability) of CD4^+^ T cells, CD8^+^ T cells and NK cells were both remarkably increased in survivors, as well as counts of IFN-γ^+^CD4^+^ T cells, IFN-γ^+^CD8^+^ T cells and IFN-γ^+^ NK cells. Univariable analyses showed that these immunology parameters were significantly different between these 2 groups (Table 1). As shown in Supplementary Table 2, these immunological parameters still had significant difference between the matched groups after propensity-score-matched analysis, except for the function of CD8^+^ T cells and the number and function of NK cells.

### Performance of IFN-γ^+^CD4^+^ T Cell Number in Predicting Prognosis

**Figure 3:**
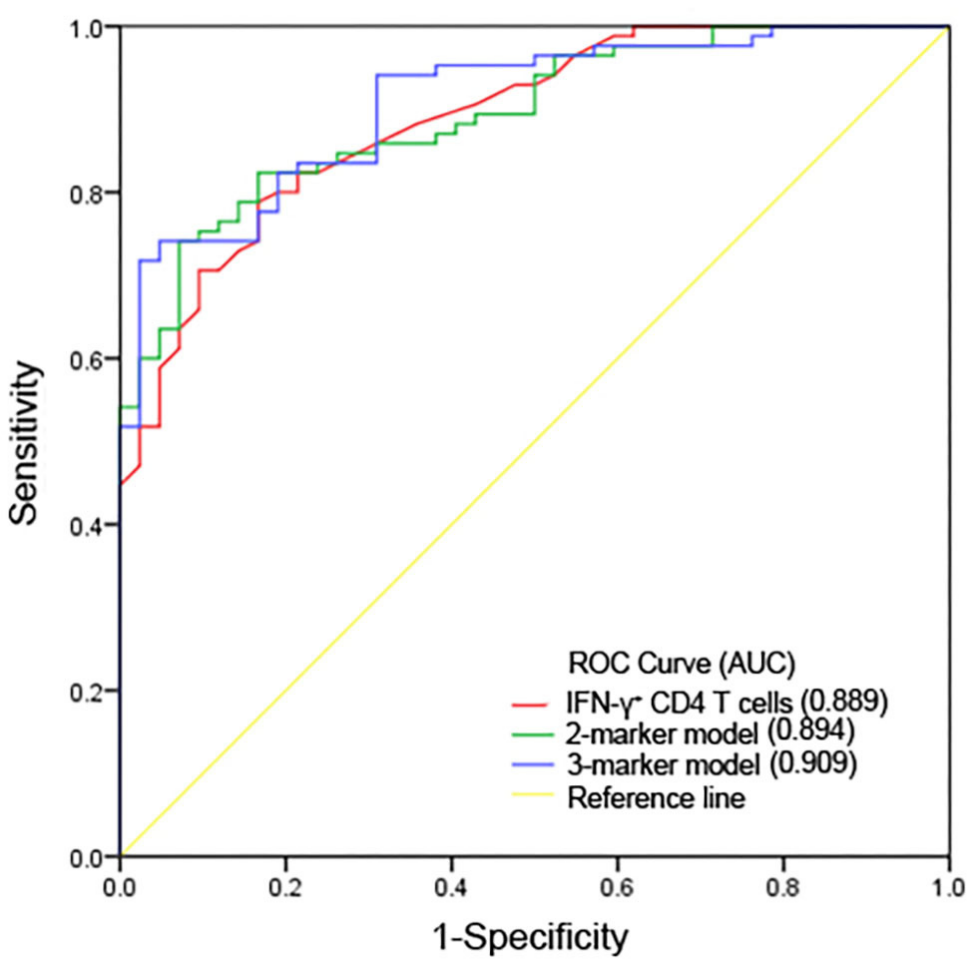
ROC curves for predicting ability for 30-day mortality of IFN-γ^+^ CD4^+^ T cell numbers and predictive models. AUC, area under the curve.

**Figure 4:**
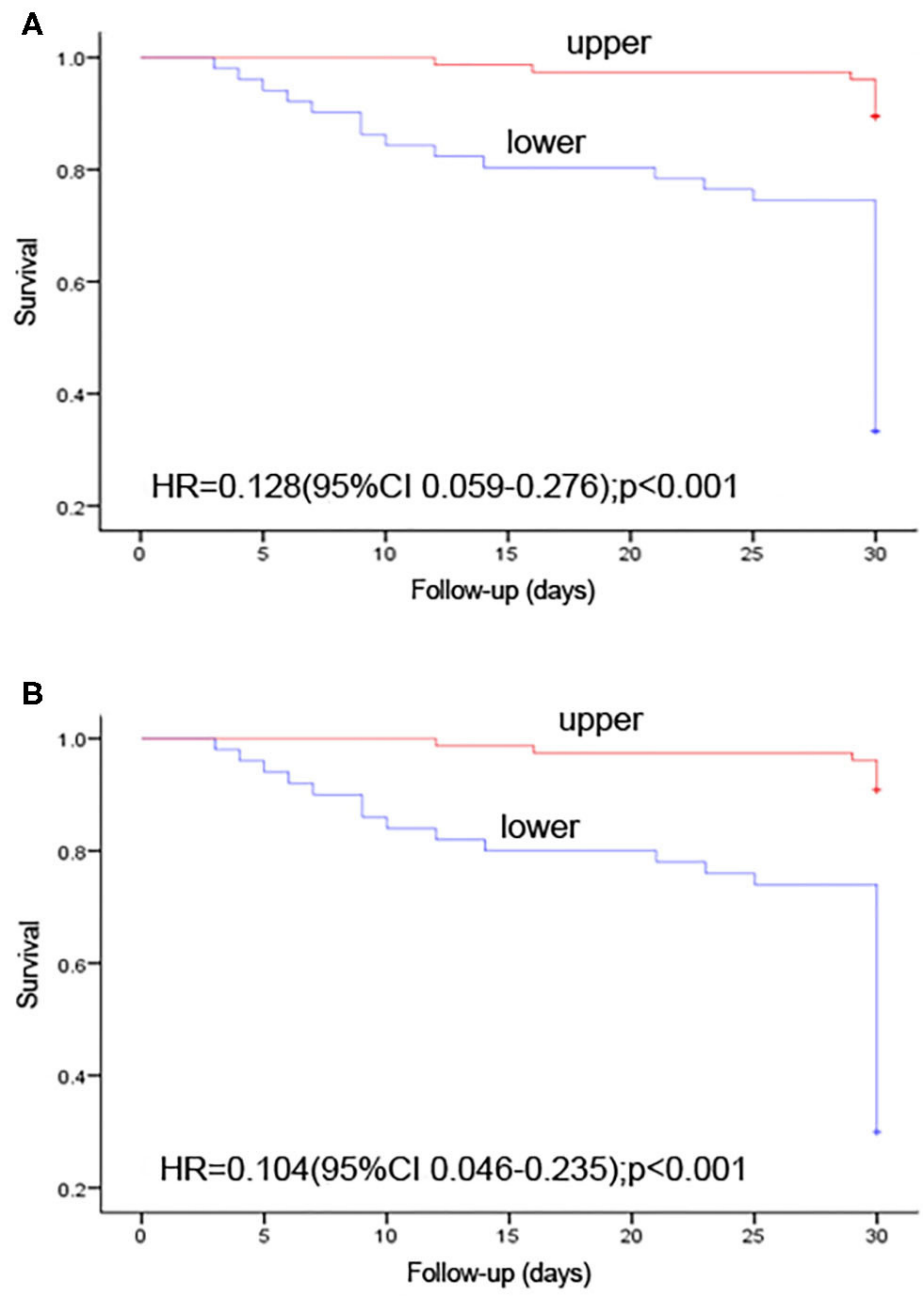
Kaplan-Meier survival curves showing the impact on 30-day mortality. **(A)** number of IFN-γ^+^ CD4^+^ T cells per microliter > 39.5 vs. <39.5; **(B)** *P*-value of 2-marker predictive model ≥ 0.593 vs. <0.593. +, right censoring of data.

The survivor group had an approximately 4-fold increase in IFN-γ^+^CD4^+^ T cell number vs. non-survivors. After propensity-score-matching, our data indicated that IFN-γ^+^CD4^+^ T cell number impacted the prognosis of CRO infection independently (Supplementary Table 2). Among immunology parameters, odds ratio (OR) of IFN-γ^+^CD4^+^ T cell number was the highest (OR, 17.000; 95% CI, 6.669 −43.335; *P* < 0.001; Table 2), namely, IFN-γ^+^CD4^+^ T cell number was better than other parameters in predicting patients' prognosis. We further conducted ROC analysis to evaluate its performance and the AUC was 0.889 (95% CI, 0.834–0.945) (Figure 3). When the cut-off value was set at 39.50, the sensitivity, specificity, and accuracy were 81.0, 80.0, and 80.3%, respectively (Table 3). Kaplan-Meier survival curves are shown in Figure 4A and indicated that mortality in patients with IFN-γ^+^ CD4^+^ T cell number <39.50/μl was significantly higher than that in patients with IFN-γ^+^ CD4^+^ T cell number > 39.5/μl.

**Table 2:** Univariate and multivariate analyses of risk factors associated with 30-day mortality of CRO-infected patients.

	**Univariate analysis**			**2-marker model**			**3-marker model**		
	***P*-Value**	**OR**	**95% CI**	***P*-Value**	**aOR**	**95% CI**	***P*-Value**	**aOR**	**95% CI**
Neurologic disease	<0.001	4.082	1.869–8.929						
Renal disease	0.012	0.348	0.151–0.803						
Renal replacement therapy	<0.001	0.206	0.093–0.455						
Multiple organ failure	<0.001	0.209	0.094–0.463				0.016	0.280	0.099–0.790
WBC counts (× 10^9^/L)	0.002	0.159	0.057–0.445						
Neutrophils proportion (%)	<0.001	0.145	0.063–0.338						
Lymphocyte counts (× 10^9^/L)	<0.001	10.526	4.348–25.641						
Platelet counts (× 10^9^/L)	<0.001	8.475	3.367–21.277						
AST (U/L)	0.030	0.219	0.092–0.520						
Creatinine (μmol/L)	0.010	0.278	0.128–0.605	0.073	0.994	0.988–1.001	0.087	0.994	0.988–1.001
CD4^+^ T cells (/μl)	<0.001	1 <0.001	4.255–23.256						
CD8^+^ T cells (/μl)	<0.001	10.526	4.348–25.641						
NK cells (/μl)	<0.001	8.130	3.472–19.231						
IFN-γ^+^ CD4^+^ T cells (%)	<0.001	8.475	3.367–21.277						
IFN-γ^+^ CD8^+^ T cells (%)	0.023	2.558	1.198–5.464						
IFN-γ^+^ NK cells (%)	0.028	9.346	2.096–41.667						
IFN-γ^+^ CD4^+^ T cells (/μl)	<0.001	17.000	6.669–43.335	<0.001	1.059	1.035–1.084	<0.001	1.056	1.031–1.082
IFN-γ^+^ CD8^+^ T cells (/μl)	<0.001	8.197	3.367-2 <0.001						
IFN-γ^+^ NK cells (/μl)	<0.001	8.772	3.676–21.277						

*WBC, white blood cells; AST, aspartate aminotransferase; IFN-γ, interferon-γ; NK, Natural killer cell; OR, odds ratio; CI, confidence interval; aOR, adjusted odds ratio*.

**Table 3:** The performance of IFN-γ^+^ CD4^+^ T cell numbers and predictive models in predicting prognosis.

	**Value (95% CI)**		
**Variable**	**IFN-**γ^+^**CD4^**+**^**	**2-marker model**	**3-marker model**
	**T cell numbers**		
AUC	0.889 (0.834–0.945)	0.894 (0.841–0.947)	0.909 (0.859–0.958)
Cut-off value	39.50	0.593	0.606
Sensitivity (%)	0.810	0.833	0.810
Specificity (%)	0.800	0.824	0.824
PPV (%)	0.667	0.700	0.694
NPV (%)	0.895	0.909	0.897
Accuracy (%)	0.803	0.827	0.819

*CI, confidence interval; IFN-γ, interferon-γ; AUC, area under the curve; PPV, positive predictive value; NPV, negative predictive value*.

### Development and Performance of Predictive Models

To predict prognosis objectively and fleetly, we took laboratory parameters with statistical significance (*P* < 0.05) in univariate analysis for further multivariable analysis. It was noted that there was significant correlation between immunological parameters, as well as some biological parameters. Finally, 7 laboratory parameters, including WBC counts, platelet counts, AST concentration, creatinine concentration, and numbers of IFN-γ^+^CD4^+^ T cell, IFN-γ^+^CD8^+^ T cell and IFN-γ^+^ NK cell, were selected as candidates for further binary logistic regression analysis. In multivariate analysis, IFN-γ^+^CD4^+^ T cell number (adjusted odds ratio [aOR], 1.059; 95% CI, 1.035 −1.084; *P* < 0.001) and creatinine concentration (aOR, 0.994; 95% CI, 0.988–1.001; *P* = 0.073) were independently associated with 30-day mortality (Table 2). Thereinto, IFN-γ^+^CD4^+^ T cell number was beneficial to survival, whereas creatinine concentration was harmful. Based on regression coefficients, we set up a mathematical equation as follows to predict 30-day survival of CRO-infected patients. The score for each patient was calculated, and a higher score would predict a greater likelihood of survival. ROC analysis showed that the AUC of the 2-marker model was 0.894 (95% CI, 0.841–0.947) (Figure 3). The sensitivity, specificity and accuracy, with a cut-off level of 0.593, were 83.3, 82.4, and 82.7%, respectively (Table 3). Kaplan-Meier survival analysis displayed that patients with score > 0.593 had a significantly higher 30-day survival rate than those with score <0.593 (*P* < 0.001; Figure 4B).

$$\text{P}=1/\left[1+{\text{e}}^{-\left(-1.392+0.057\times \text{IFN}-\gamma^{+}\text{CD}4^{+}\text{T cell number}-0.006\times \text{Cr}\right)}\right]$$

P, predictive value; e, natural logarithm; IFN-γ^+^ CD4^+^T cell number, number of IFN-γ^+^ CD4^+^ T cells per microliter; Cr, creatinine concentration(μmol/L).

We also conducted a binary logistic regression analysis of valuable laboratory and clinical parameters. In multivariate analysis, IFN-γ^+^CD4^+^ T cell number (aOR, 1.056; 95% CI, 1.031 to 1.082; *P* < 0.001), creatinine concentration (aOR, 0.994; 95% CI, 0.988 −1.001; *P* = 0.087) and MOF (aOR, 0.280; 95% CI, 0.099 −0.790; *P* = 0.016) (Table 2) were selected as predictive model markers. Thereinto, MOF was prejudicial to survival, and the marker was given a score of 1 if present and 0 if absent. Furthermore, we analyzed the performance of the model and its AUC was 0.909 (95% CI, 0.859–0.958) (Figure 3). When the cut-off value was set at 0.606, the sensitivity, specificity and accuracy were 81.0, 82.4, and 81.9%, respectively (Table 3).

$$\begin{array}{l} \text{P}=1/[1\\ \;+{\text{e}}^{-\left(-0.636+0.055\times \text{IFN}-\gamma^{+}\text{CD}4^{+}\text{T cell number}-0.006\times \text{Cr}-1.273\times \text{MOF}\right)]} \end{array}$$

P, predictive value; e, natural logarithm; IFN-γ^+^ CD4^+^T cell number, number of IFN-γ^+^ CD4^+^ T cells per microliter; Cr, creatinine concentration(μmol/L); MOF, multiple organ failure.

### Potential Value of IFN-γ^+^CD4^+^ T Cell Number and Predictive Models in Prognosis Monitor

We collected 6 continually infected cases that the time interval of positive cultures was 30 days or more. Demographic and clinical characteristics of these patients are shown as Supplementary Table 1. The count of IFN-γ^+^CD4^+^ T cells and quantitative value of predictive models changed during patients' therapy, and their variation trends were roughly consistent with APACHE II score. The result indicated that IFN-γ^+^CD4^+^ T cell number and predictive models could reflect therapeutic efficacy, which suggested they had potential value in monitoring patients' prognosis.

## Discussion

Due to rapidly mobile genetic elements and limited effective treatment strategies, carbapenem resistance transmit between manifold gram-negative bacteria and adversely affect patients' clinical outcomes, resulted in significant morbidity and mortality (Falagas et al., 2014; Lemos et al., 2014; Zhang et al., 2016; Tamma et al., 2017; Agyeman et al., 2020). CROs have caused several outbreaks and become a global public health issue (Yu et al., 2017; Ben-Chetrit et al., 2018; Gu et al., 2018). Although studies on risk factors and clinical outcomes for CRO infection were numerous, rare studies used these markers to establish models to predict prognosis. Meanwhile, there was no research regarding lymphocyte function in prognosis of CRO infection yet. In the present study, we analyzed lymphocyte number and function in patients with different outcomes, and successfully established mathematical models to predict the prognosis of CRO-infected individuals through combinations of laboratory markers and clinical factors. To our knowledge, this is the first report to use immunity model to predict the prognosis of CRO infection.

The 30-day mortality rate was 33.07% in our study. In accordance with our results, Agyeman AA et al. reported that the pooled mortality was 37.2% in their meta-analysis which involved 54 studies and 3,195 CRKP-infected patients; and Lemos EV reported 850 deaths (33%) among 2,546 CRAB-infected patients in their meta-analysis including 16 observational studies (Lemos et al., 2014; Agyeman et al., 2020). In our study, univariate analyses showed that some clinical and laboratory variables were related to outcomes of CRO-infected patients. Thereinto, the occurrence of renal disease was harmful to prognosis, therefore renal replacement therapy was much more frequent among non-survivors, which is consistent with previous report (Shields et al., 2018). Our study also illustrated that MOF was associated with 30-day mortality, which was similar to previous findings (Niu et al., 2018). Among routine biological parameters, WBC counts, neutrophil proportion and lymphocyte counts were the most common indices used for auxiliary diagnosis of infectious diseases. Regarding platelet counts, it is reported that platelets play a pivotal role in the immunomodulatory process and that abnormal platelet count is a marker of poor prognosis in critically ill patients (Hui et al., 2011; Vinholt et al., 2016; Oh et al., 2017). There were statistical differences between survivors and non-survivors in terms of AST and creatinine, suggesting that impairment of liver or renal function is a related factor of poor prognosis in CRO-infected patients.

Among CRO-infected patients, previous studies revealed that higher mortality rate has been observed in immunosuppressed individuals, including the presence of hematological malignancies, steroid therapy or other immunosuppressive therapy (Huang et al., 2012; Wang et al., 2016). Our group has previously reported that both the number and function of lymphocytes are decreased in patients with immunosuppressive conditions (Luo et al., 2019). Combination of lymphocyte number and function can potentially evaluate host immunity, which suggests that evaluation of host immunity plays an important role in monitoring and prognosis of infectious diseases (Boomer et al., 2012; Spec et al., 2016). In our study, non-survivor group was lower than the survivor group in all immunological parameters, suggesting that host immunity had an influence on the outcomes of CRO-infected patients. Lots of studies indicated that in the early stage of sepsis induced by different pathogens, circulating T lymphocytes were decreased to varying degrees in number and function of them, manifested as expression of typical T cell exhaustion markers, programmed death-1 (PD-1) or its ligand (PD-L1) (Gogos et al., 2010; Spec et al., 2016). Further researches have shown that PD-1 receptor system constitutes an immunoregulatory pathway that negatively controls immune responses, and that PD-1 levels correlate with increased mortality, nosocomial infection and immune dysfunctions in septic patients (Guignant et al., 2011; Boomer et al., 2012). Among all immune parameters of our study, IFN-γ^+^CD4^+^ T cell number had the best performance in the prediction of patients' prognosis. What's more, it could reflect the change of immune status during therapy, which suggests it has the potential in monitoring patients' prognosis. Precision immunotherapy has been shown to be beneficial to the treatment and prognosis of sepsis patients (Spruijt et al., 2010; Hutchins et al., 2014). We did observe that the severe impairment of CD4^+^ T cells in the early period of CRO infection was correlated with death, therefore, therapy that reverses T cell exhaustion may restore host immunity of patients and improve their survival.

Although many parameters discussed above were associated with 30-day mortality, the value of using a single parameter in predicting prognosis is very limited in clinical practice because of low sensitivity or specificity. Thus, combination of the selected valuable markers to establish a mathematical model may help to solve this problem. Given that all immunological markers were valuable and congenerous, we screened the most effective markers by OR value. By using the logistic regression to analyze valuable laboratory parameters, we have successfully established the 2-marker model, with a sensitivity of 83.3% and a specificity of 82.4%. Laboratory markers selected in this model are IFN-γ^+^CD4^+^ T cell number and creatinine concentration, and IFN-γ^+^CD4^+^ T cell number was the stronger predictor based on regression coefficients. The 2 markers are inexpensive and easily available in routine practice. Abnormal serum creatinine is associated with renal dysfunction, and acute kidney injury is a common complication of critical illness that carries high mortality rates (Karvellas et al., 2011; Yang et al., 2017). The 2-marker model is meaningful in clinical practice, as this model has effectively predictive performance and can be applied in the early phase of CRO-infection Subsequently, we established a 3-marker predictive model, which included the clinical factor MOF except for above 2 markers. MOF may be caused by an early over-reaction of the immune system or a late immune paralysis, and is the common cause of late death in infection patients (Spruijt et al., 2010; Wu et al., 2019). ROC analysis showed good predictive accuracy of this 3-marker model.

Several limitations should be mentioned in our study. First, this is a single-center design with a small proportion of patients and limited infection types. The limited number of patients did not allow us to develop a single and comprehensive multivariate model that included all potential variables influencing mortality, which could generate bias in the model establishment and limits these typical findings' use in clinical practice. Therefore, future validation studies in multiple centers with large numbers of patients and various infection types are necessary to verify variables that were not included in the multivariate analysis. Secondly, although the combination of lymphocyte number and function was analyzed, other immunological markers were not evaluated in our study. Moreover, the exact survival time of some patients was missed, and the temporal relationship between risk-factors and mortality was not analyzed. In the future, a Cox regression model could be made to study whether the number of IFN-γ^+^CD4^+^ T cells is a good predictor of time to death for CRO infection. Thirdly, the use of immunosuppressive agents could affect the results of immunological parameters. Our data showed that the number of IFN-γ^+^CD4^+^ T cells in immunosuppressive agent-unused group was slightly higher than in immunosuppressive agent-used group in both survivors and non-survivors, but this did not achieve significant difference (Supplementary Figure 2). Finally, as samples were obtained at only a single time point (infection), we were unable to determine the changes of immune status that occurred throughout the course of illness. Dynamic tracking of immune function in certain patients is a better way to evaluate the performance of immune markers and predictive model, and will be more helpful to guide individualized treatment and improve prognosis.

In conclusion, this study demonstrated that impaired lymphocyte function was a critical factor to influence individual outcomes in patients with CRO infection and that IFN-γ^+^CD4^+^ T cell number could indicate outcome well, as well as had potential value in monitoring prognosis. By combining laboratory parameters with clinical factors, we successfully established two models that not only showed good performance in predicting prognosis of CRO-infected patients but also had potential value in monitoring prognosis during therapy. These results might provide a useful tool to guide individualized therapy and improve prognosis in clinical practice.

## Data Availability Statement

All datasets generated for this study are included in the article/Supplementary Material.

## Ethics Statement

The studies involving human participants were reviewed and approved by the ethical committee of Tongji hospital, Tongji Medical College, Huazhong University of Science and Technology. The patients/participants provided their written informed consent to participate in this study.

## Author Contributions

QL, YL, and GT performed experiments. YW, SL, and YZ managed participant recruitment and clinical data collection. LM and WL analyzed data. QL wrote the paper. FW and ZS designed the study. All authors read and approved the final manuscript.

## Conflict of Interest

The authors declare that the research was conducted in the absence of any commercial or financial relationships that could be construed as a potential conflict of interest.
