# Supplementary material for: The Effect of Host Immunity on Predicting the Mortality of Carbapenem-Resistant Organism Infection
Source: Front Cell Infect Microbiol. 2020 Sep 10;10:480. doi: 10.3389/fcimb.2020.00480 (PMC7533642; doi:10.3389/fcimb.2020.00480)
Supplement: Supplementary file 1 [file Data_Sheet_1.PDF]

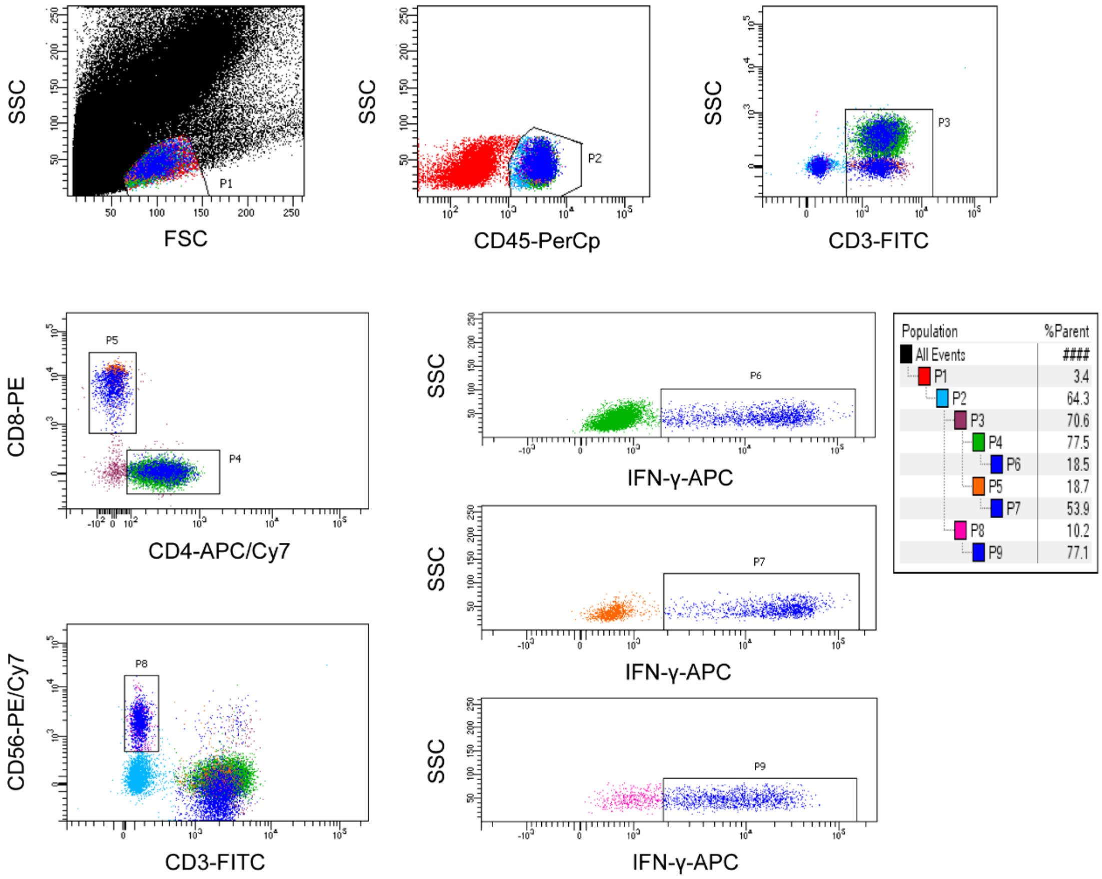

Supplementary Figure 1. The final flow analysis template of lymphocyte function assay. Representative flow plots showing the gating strategies of IFN-γ^+ cells in $CD4^+$, $CD8^+$ T cells, and NK cells. The percentage of IFN-γ^+ cells in $CD3^+CD4^+CD8^-$ cells was regarded as the function of $CD4^+$ T cells; the percentage of IFN-γ^+ cells in $CD3^+CD4^-CD8^+$ cells was regarded as the function of $CD8^+$ T cells; the percentage of IFN-γ^+ cells in $CD3^-CD56^+$ cells was regarded as the function of NK cells.

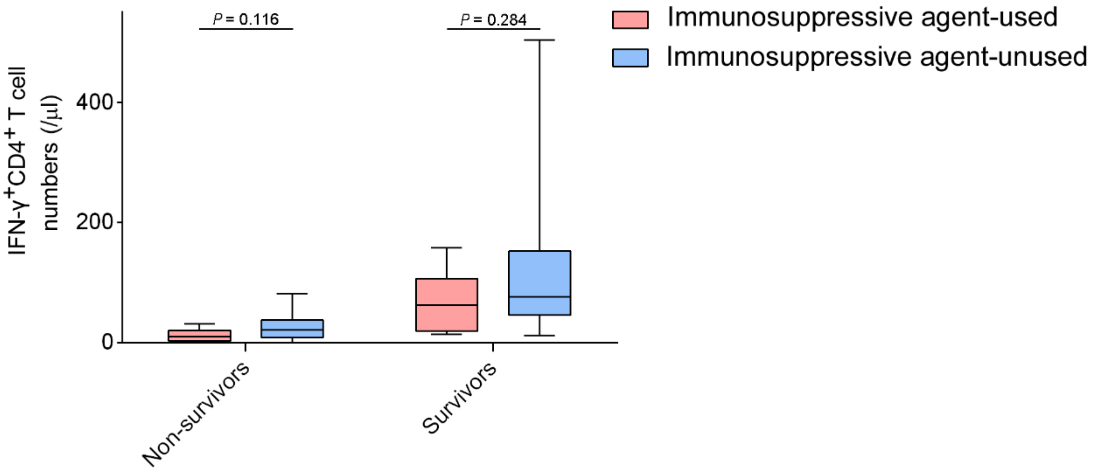

Supplementary Figure 2. The number of IFN-γ^{+}CD4^{+} T cells stratified by immunosuppressive therapy in survivors and non-survivors.
