# Supplementary material for: The Effect of Host Immunity on Predicting the Mortality of Carbapenem-Resistant Organism Infection
Source: Front Cell Infect Microbiol. 2020 Sep 10;10:480. doi: 10.3389/fcimb.2020.00480 (PMC7533642; doi:10.3389/fcimb.2020.00480)
Supplement: Supplementary file 2 [file Data_Sheet_2.docx]

Supplementary Table 1. Demographic and clinical characteristics of patients continually infected with CRO.

| Patient number | Gender | Age (years) | Ward | Date of culture | Culture sources | Pathogen | IFN-γ^+^ CD4^+^ T cells (/μl) | 2-marker Model | 3-marker Model | 30-day  outcome | APAC-HE II |
| --- | --- | --- | --- | --- | --- | --- | --- | --- | --- | --- | --- |
| 1 | M | 50 | ICU | 2019/1/7 | BAL | CRKP | 320.98 | 1.000 | 1.000 | S | 13 |
|  |  |  | ICU | 2019/3/17 | Sputum | CRKP | 503.25 | 1.000 | 1.000 | S | 17 |
| 2 | M | 53 | ICU | 2019/4/22 | Blood | CRKP | 127.31 | 0.995 | 0.997 | S | 22 |
|  |  |  | ICU | 2019/6/7 | Blood | CRKP | 72.78 | 0.909 | 0.949 | S | 23 |
| 3 | M | 49 | ICU | 2019/5/13 | BAL | CRKP | 202.12 | 1.000 | 1.000 | S | 8 |
|  |  |  | Medical | 2019/6/5 | Blood | CRKP | 162.49 | 0.999 | 1.000 | S | 8 |
| 4 | M | 23 | Medical | 2019/5/18 | Sputum | CRAB | 54.68 | 0.805 | 0.688 | S | 15 |
|  |  |  | Medical | 2019/7/1 | Sputum | CRKP | 103.55 | 0.987 | 0.974 | S | 25 |
|  |  |  | Medical | 2019/8/2 | Sputum | CRKP | 172.73 | 1.000 | 0.999 | S | 21 |
| 5 | F | 51 | Medical | 2019/7/2 | BAL | CRAB | 59.86 | 0.852 | 0.916 | S | 8 |
|  |  |  | Medical | 2019/8/5 | BAL | CRAB | 67.21 | 0.857 | 0.918 | S | 7 |
| 6 | M | 63 | Medical | 2019/7/18 | Sputum | CRAB | 10.56 | 0.246 | 0.405 | S | 8 |
|  |  |  | Medical | 2019/8/27 | Sputum | CRAB | 7.78 | 0.192 | 0.123 | N | 5 |

Abbreviations: M, male; F, female; ICU, intensive care unit; BAL, Broncho-alveolar lavage; CRKP, carbapenem-resistant *Klebsiella pneumoniae*; CRAB, carbapenem-resistant *Acinetobacter baumannii*; S, survivor; N, non-survivor; APACHE II, Acute Physiology and Chronic Health Evaluation II.

Supplementary Table 2. Immunological parameters of CRO-infected patients after propensity score matching.

| Immunology parameters | Survivors(n=35) | Non-survivors(n=24) | *P* Value |
| --- | --- | --- | --- |
| CD4+ T cells (/μl) | 463.00 (311.00-687.00) | 154.00 (70.750-298.50) | <0.001 |
| CD8+ T cells (/μl) | 237.00 (144.00-492.00) | 82.50 (56.75-150.75) | 0.001 |
| NK cells (/μl) | 90.00 (41.00-169.00) | 24.50 (15.25-58.75) | 0.125 |
| IFN-γ+ CD4+ T cells (%) | 16.90 (14.16-32.30) | 14.85 (9.22-20.77) | 0.015 |
| IFN-γ+ CD8+ T cells (%) | 49.90 (29.02-66.31) | 46.92 (26.23-65.08) | 0.726 |
| IFN-γ+ NK cells (%) | 51.88 (31.35-80.59) | 47.46 (25.41-72.00) | 0.348 |
| IFN-γ+ CD4+ T cells (/μl) | 76.00 (38.00-152.00) | 20.50 (8.25-36.75) | <0.001 |
| IFN-γ+ CD8+ T cells (/μl) | 88.00 (41.00-196.00) | 36.00 (21.00-72.00) | 0.004 |
| IFN-γ+ NK cells (/μl) | 38.00 (13.00-73.00) | 12.00(5.00-26.25) | 0.001 |

Data are presented as medians (25th-75th centiles).
